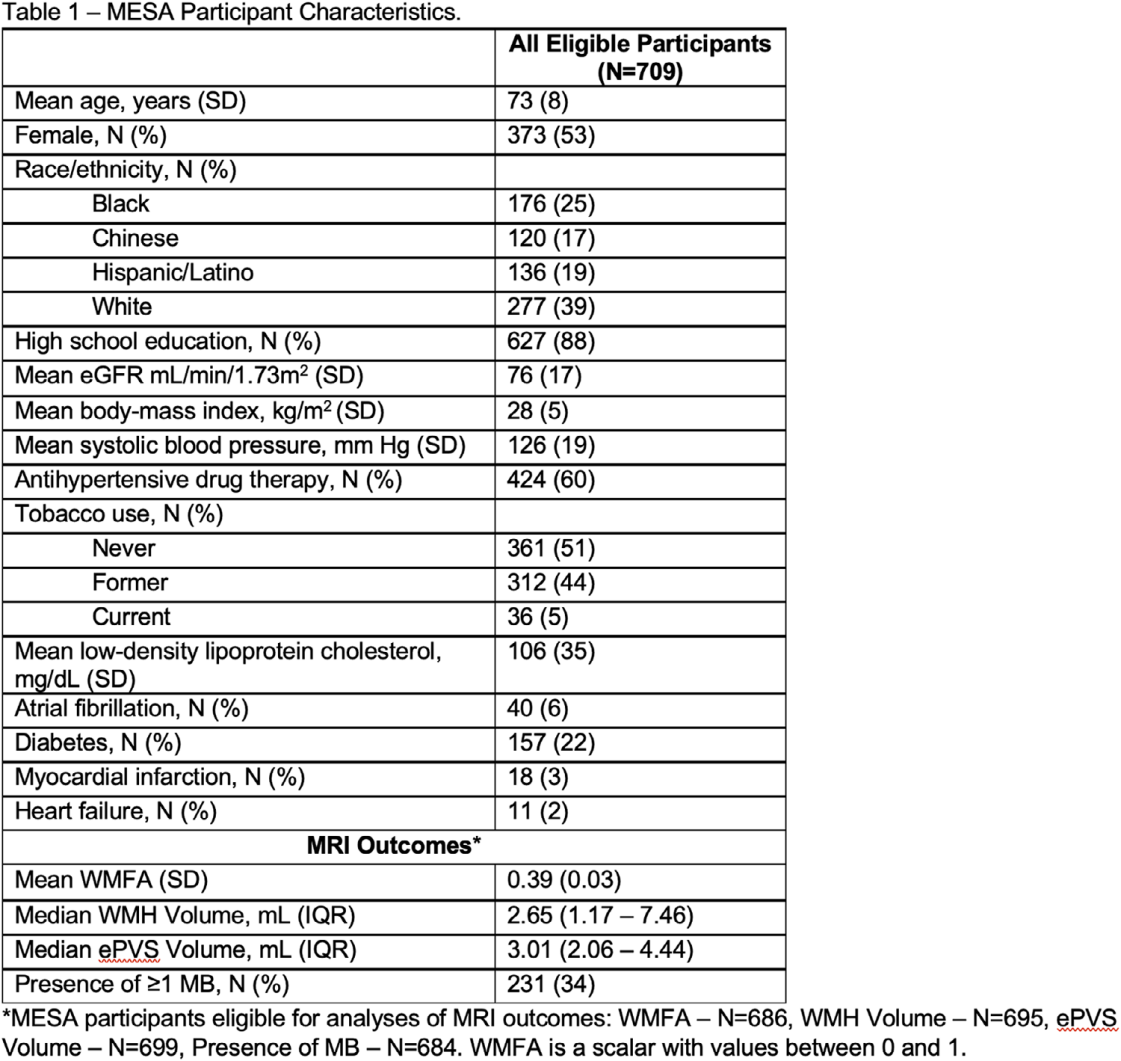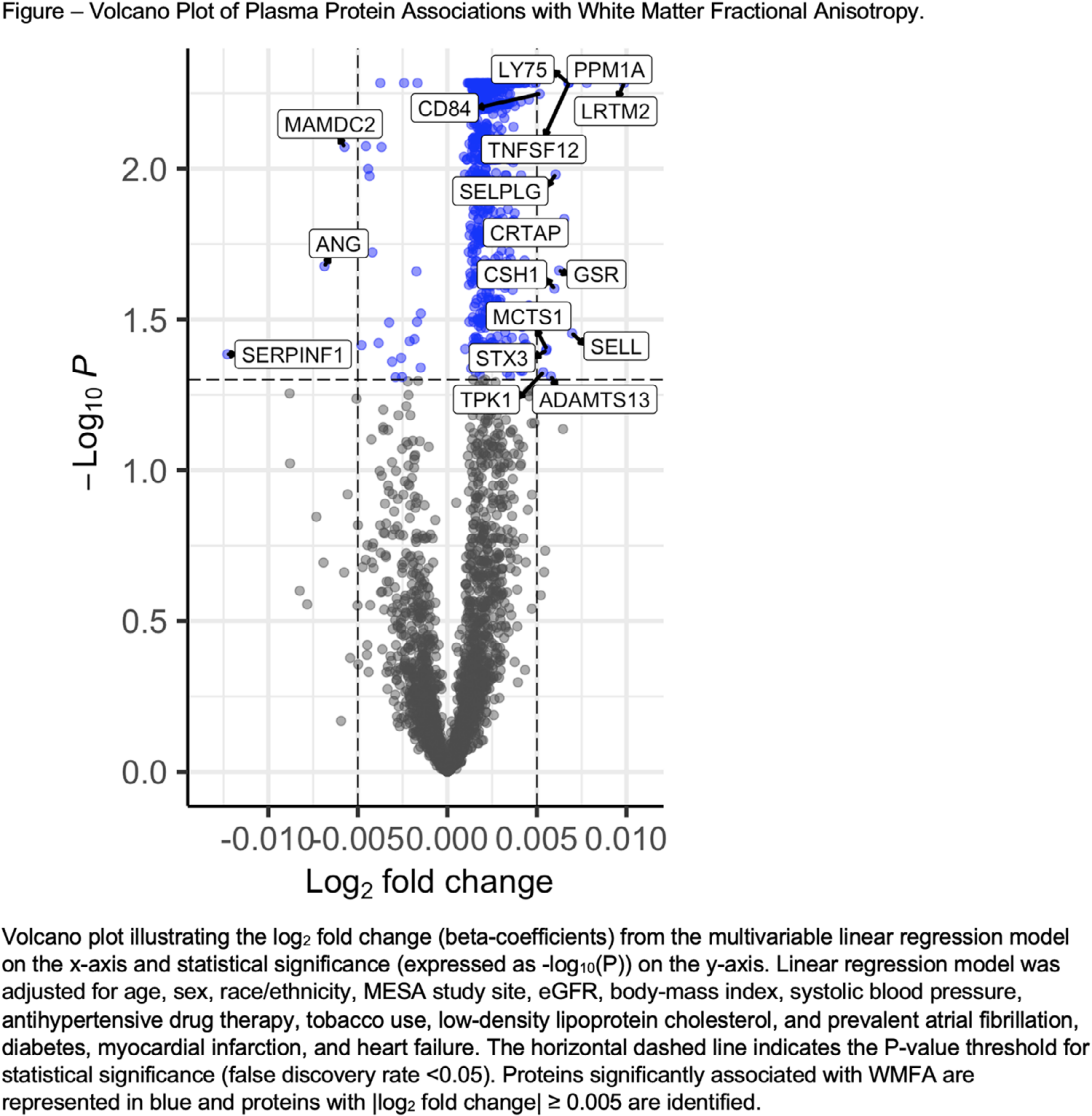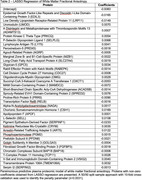# Plasma Proteomic Determinants of Small Vessel Disease of the Brain: the Multi‐Ethnic Study of Atherosclerosis

**DOI:** 10.1002/alz.084577

**Published:** 2025-01-09

**Authors:** Rizwan Kalani, Alison E Fohner, Thomas R. Austin, Sheina Emrani, Paul N. Jensen, Alexis Frazier‐Wood, Alain G. Bertoni, Sanjiv J. Shah, Mohamad Habes, Tanweer Rashid, Sokratis Charisis, Keenan A. Walker, William T Longstreth, David L Tirschwell, Bruce M. Psaty, James Floyd, Usman A Tahir, Robert Edgardo Gerszten, Jerome I Rotter, Stephen S Rich, Susan R. Heckbert, Timothy M. Hughes

**Affiliations:** ^1^ University of Washington, Seattle, WA USA; ^2^ Department of Neurology, Perelman School of Medicine, University of Pennsylvania, Philadelphia, PA USA; ^3^ Baylor College of Medicine, Houston, TX USA; ^4^ Wake Forest School of Medicine, Winston‐Salem, NC USA; ^5^ Northwestern University Feinberg School of Medicine, Chicago, IL USA; ^6^ Glenn Biggs Institute for Alzheimer’s & Neurodegenerative Diseases, University of Texas Health Sciences Center at San Antonio, San Antonio, TX USA; ^7^ Neuroimage Analytics Laboratory (NAL) and the Biggs Institute Neuroimaging Core (BINC), Glenn Biggs Institute for Alzheimer’s & Neurodegenerative Diseases, University of Texas Health Sciences Center, San Antonio, TX USA; ^8^ Laboratory of Behavioral Neuroscience, National Institute on Aging, Intramural Research Program, Baltimore, MD USA; ^9^ Beth Israel Deconess Medical Center, Boston, MA USA; ^10^ Beth Israel Deaconess Medical Center, Boston, MA USA; ^11^ The Institute for Translational Genomics and Population Sciences, The Lundquist Institute for Biomedical Innovation at Harbor‐UCLA Medical Center, Torrance, CA USA; ^12^ Center for Public Health Genomics, University of Virginia School of Medicine, Charlottesville, VA USA; ^13^ Wake Forest University School of Medicine, Winston‐Salem, NC USA

## Abstract

**Background:**

The identification of novel blood‐based biomarkers of small vessel disease of the brain (SVD) may improve pathophysiologic understanding and inform the development of new therapeutic strategies for prevention. We evaluated plasma proteomic associations of white matter fractional anisotropy (WMFA), white matter hyperintensity (WMH) volume, enlarged perivascular space (ePVS) volume, and the presence of microbleeds (MB) on brain magnetic resonance imaging (MRI) in the population‐based Multi‐Ethnic Study of Atherosclerosis (MESA).

**Methods:**

Eligible MESA participants had 2941 plasma proteins measured from stored blood samples (collected in 2016‐2018) using the antibody‐based Olink proteomics platform, and completed brain MRI scans in 2018‐2019. Participants with quality control exclusion of protein measurements, missing covariate data, and poor quality or missing MRI outcome variables were excluded. The cross‐sectional association between the abundance of each plasma protein (normalized protein expression – a relative protein quantification unit measured on a log_2_ scale) was modeled separately with WMFA, WMH volume, total ePVS volume, and the presence of MBs using multivariable linear or modified Poisson regression, adjusting for demographic variables, estimated glomerular filtration rate (eGFR), and SVD risk factors. The Benjamini‐Hochberg procedure was used to control the false discovery rate (FDR) <0.05 to account for multiple hypothesis testing. For proteins independently associated with the SVD markers on MRI, penalized regression with least absolute shrinkage and selection operator (LASSO) was used to create a parsimonious proteomic model.

**Results:**

Eligible participants (total N=709) had a mean age of 73 years, 53% were women, 25% were Black, 17% were Chinese, 19% were Hispanic or Latino, and 39% were White (Table 1). After adjustment for demographics, eGFR, and SVD risk factors, 769 plasma proteins were associated with WMFA (FDR <0.05) (Figure). LASSO regression identified a 37‐protein model predictive of WMFA (Table 2). We did not find plasma proteins to be independently associated with WMH volume, ePVS volume, or the presence of MBs.

**Conclusion:**

Multiple circulating proteins – implicated in central nervous system myelination, lipid metabolism, angiogenesis, coagulation, cellular adhesion and migration, appetite regulation, energy homeostasis, systemic inflammation, and immune regulation – were independently associated with WMFA in a multi‐ethnic cohort of older adults.